# The role of trained immunity in sepsis

**DOI:** 10.3389/fimmu.2024.1449986

**Published:** 2024-08-15

**Authors:** Wenjuan Wang, Lisi Ma, Bin Liu, Liangliang Ouyang

**Affiliations:** Department of Medical Laboratory, Affiliated Hospital of Jiujiang University, Jiujiang, Jiangxi, China

**Keywords:** sepsis, immunotherapy, innate immune cells, trained immunity, reprogramming

## Abstract

Sepsis is defined as a life-threatening organ dysfunction syndrome caused by dysregulated host response to infection, characterized by a systemic inflammatory response to infection. The use of antibiotics, fluid resuscitation, and organ support therapy has limited prognostic benefit in patients with sepsis, and its incidence is not diminishing, which is attracting increased attention in medicine. Sepsis remains one of the most debilitating and expensive illnesses. One of the main reasons of septic mortality is now understood to be disruption of immune homeostasis. Immunotherapy is revolutionizing the treatment of illnesses in which dysregulated immune responses play a significant role. This “trained immunity”, which is a potent defense against infection regardless of the type of bacteria, fungus, or virus, is attributed to the discovery that the innate immune cells possess immune memory via metabolic and epigenetic reprogramming. Here we reviewed the immunotherapy of innate immune cells in sepsis, the features of trained immunity, and the relationship between trained immunity and sepsis.

## 1 Introduction

Sepsis is a clinical syndrome that is thought to be a competition between the pathogen and the host immune system. The terms sepsis and septic shock, which are defined as life-threatening organ dysfunction brought on by a dysregulated host response to an infection, have been updated by the third sepsis consensus conference (1). sepsis morbidity remains persistently high, and the World Health Organization (WHO) considers it as one of the global health priority questions. With the advancement of medical equipment, procedures, and techniques, sepsis mortality has gradually declined; however, there is still much mortality and room for improvement, and sepsis is one of the primary causes of death for critically ill patients in the intensive care unit (ICU) (2). Sepsis had a 20.6% fatality rate, according to an epidemiological study report (3). Furthermore, studies have shown an increased risk of cardiovascular events, neurocognitive dysfunction, and developing dementia even after the patient has recovered (4–6). At the onset of a sepsis episode, hyperinflammation and immunosuppression are concurrent rather than sequential phenomena (7). The previously held perspective that immunosuppression is succeeded by hyperinflammation at a later stage is no longer considered valid (8). Contemporary research indicates that the initial stages of infection are marked by the simultaneous occurrence of both pro-inflammatory and anti-inflammatory cytokine storms. The perturbation of the balance between these opposing responses ultimately dictates the development of either hyperinflammation or immunosuppression. Genome-wide expression analyses of leukocytes from sepsis patients have demonstrated that the expression of genes associated with the inflammatory response and immunosuppression is upregulated subsequent to the onset of sepsis (9). Importantly, the severity of sepsis is reflected by the increased expression levels of genes contributing to immunosuppression (9). Those who survive the acute hyperinflammatory responsive phase also remain at an increased risk of dying from immunosuppression (10–14); survivors experience a dramatic decline in quality of life and an increase in the burden on society and families; survivors frequently exhibit a persistent decline in physical activity, athleticism, and muscle strength (15). Immunotherapy, which targets the immunosuppressive state that is prevalent in sepsis patients, has attracted attention recently as due to of a better understanding of the disease’s complex pathophysiology in recent decades.

Innate immune responses, which can respond quickly to a pathogen, and adaptive immune responses, which take longer to activate but are specific, are the two main categories of host immune responses. All through the way, it was considered that immunological memory was the sole hallmark of the adaptive immune response. But a growing amount of research suggests that innate immune system activation contains adaptive characteristics, increasing the immune system’s ability to eradicate the infection in the event of a recurrence. This state of affairs, termed “trained immunity”, is innate immune memory. That is, following a pathogen challenge, certain innate immune cells, like neutrophils and monocytes, display traits of trained immunity (16). It is crucial to clarify the distinction between trained immunity and immune priming. Unlike trained immunity, which involves the nonspecific enhancement of the innate immune response against a broad range of pathogens, immune priming involves the initial encounter with antigens of specificity. Upon immune priming, following the initial stimulation, the cellular immune status does not revert to the pre-infection basal level. Upon subsequent challenge, the immune response typically shows additive or synergistic effects with the initial stimulus. In contrast, during trained immunity, upon removal of the stimulus for the first time, the cellular immune state reverts to the pre-infection level before the basal level, yet retains epigenetic modifications (17). Currently, the development of innate immune training has been shown to provide wide and lasting protection against a variety of infections for weeks to months, making training immunity a potentially effective strategy for regaining the immune response. While prior studies have reported non-specific vaccination effects on newborns that can last up to five years (18), the immunological phenotype of trained immunity has been demonstrated to remain in people for at least months and up to a year (19, 20).

Immune homeostasis imbalance, inflammation, and immunosuppression can all occur continuously or simultaneously in sepsis patients, which has a major negative impact on their quality of life and health. Trained immunity can modulate the host’s innate immune cells to enhance resistance to pathogens. Understanding the features of trained immunity will improve knowledge of host defense mechanisms and open up novel prospects for the clinical application of sepsis disease prevention and treatment. In this Review, we discuss current immunotherapy strategies for sepsis, the latest discoveries of sepsis on training immunity, and highlight their significance for the development of effective strategies for treating patients with septic shock.

## 2 Immunotherapy in sepsis

**Figure 1:**
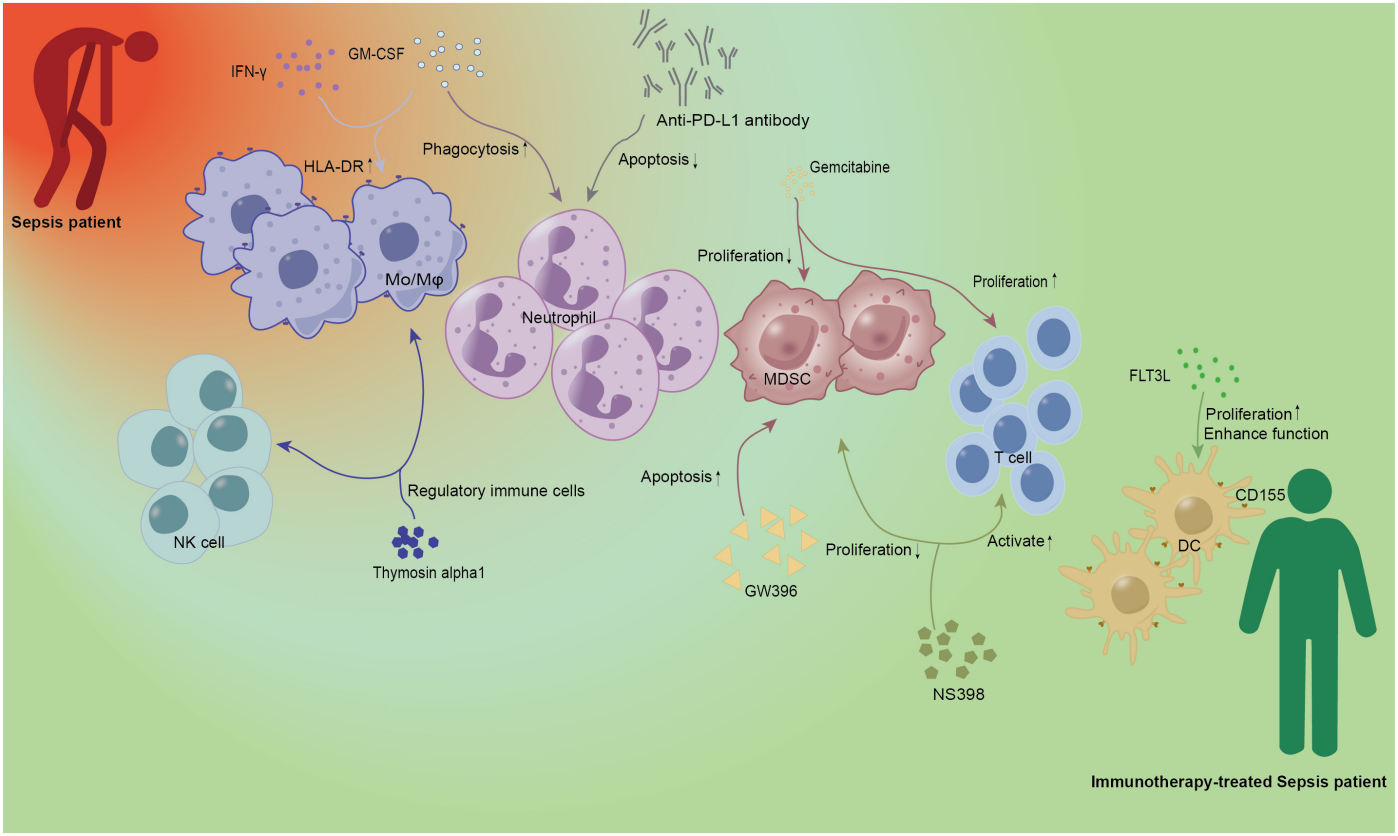
Immunotherapy of innate immune cells in sepsis. IFN-γ and GM-CSF as cytokines have been demonstrated in early studies of sepsis. IFN-γ and GM-CSF could increase the expression of HLA-DR on Mo/Mφ, and the latter could also enhance the phagocytosis of neutrophils. Anti-PD-L1 antibody can be used as Immunoglobulin to alleviate neutrophil-related apoptosis and improve the prognosis of patients with sepsis. Gemcitabine, a new cytosine nucleoside derivative, can not only play a role in anti-tumor therapy but also inhibit the proliferation of MDSC and promote the proliferation of T lymphocytes. GW396 and NS398 acted as agonists and inhibitors of the receptor, respectively, to reduce the number of MDSC and affect the activation of T cells, and regulate the immune status of sepsis. As a growth factor, FLT3L can specifically promote DC proliferation and enhance DC function. Thymosin alpha1, as an important immunomodulator, has the function of regulating immune cells. Red person, Sepsis patient; Green person, Immunotherapy-treated Sepsis patient; Mo/Mφ, monocyte/macrophage; DC, dendritic cell; NK, natural killer cell; MDSC, Myeloid-derived suppressor cell; HLA-DR, human leukocyte antigen-DR; GM-CSF, granulocyte-macrophage colony-stimulating factor; IFN-γ, interferon-γ; PD-L1, programmed death ligand 1; FLT3L, fms-like tyrosine kinase 3 ligand.

It has long been believed that an early, overactive immune inflammatory response is the primary cause of infectious mortality. Thus, diminishing inflammation in the early stages of sepsis is the main focus of many clinical trials on sepsis patients; however, this has not been shown to substantially lower sepsis mortality (21–23). In refractory sepsis and septic shock, activation of the innate immune system is a clinically desirable treatment. Several immunostimulatory therapies have demonstrated promise in preclinical or clinical trials (24–28). As shown in **Figure 1** and **Table 1**, recent studies suggest that activation of innate immune cells can be used as a treatment for sepsis.

**Table 1:** Immunotherapy of innate immune cells in sepsis.

Cell type	Species	interventions	Year	Result	References
Monocyte	Human	INF-γ	1997	Restore the deficient HLA-DR expression and *in vitro* LPS-induced TNF-α secretion	(29)
Macrophage	Human	INF-γ	2002	Restore level of HLA-DR expression in alveolar macrophages	(30)
Monocyte	Human	INF-γ	2012	Treatment with IFN-γ increased monocyte HLA-DR expression	(31)
Monocyte	Human	GM-CSF	2009	Restore level of HLA-DR expression and proinflammatory monocytic cytokine production	(25)
Monocyte	Human	GM-CSF	2018	Restore level of HLA-DR expression	(32)
Monocyte	Human	Thymosin alpha1	2013	Restore monocyte HLA-DR expression level	(33)
Neutrophil	Mouse	Anti-PD-L1 antibody	2015	Inhibit lymphocyte apoptosis	(34)
Neutrophil	Human	GM-CSF	2018	Improve neutrophil phagocytosis	(32)
MDSC	Human	Gemcitabine	2016	The number of granulocytic MDSCs and Tregs cells was decreased, and the ratio of effector T cells: Treg was increased	(35)
MDSC	Mouse	Liver X receptor agonist GW3965	2021	Improve the survival rate of septic mice, reduce multi-organ injury, and reduce the level of inflammatory cytokines.	(36)
MDSC	Mouse	Selective COX-2 inhibitor NS398	2022	Improve sepsis-induced immune dysfunction	(37)
NK cell	Human	Thymosin alpha1	2007	Increase the number of NK cells and improve cell immunity function in the patients with septic shock	(38)
DC	Mouse	FLT3L	2016	Increase the number of DCs and improve the function of DCs	(39)
DC	Mouse	anti-CD155 antibody	2017	Decease bacterial burden in both blood and peritoneal lavage fluid and improve survival rate in mouse sepsis models	(40)

MDSC, myeloid-derived suppressor cell; NK-cell, natural killer cell; DC, dendritic cell; IFN-γ, interferon-γ; GM-CSF, granulocyte–macrophage colony-stimulating factor; PD-L1, programmed death ligand 1; COX-2, cyclooxy-genase-2; FLT3L, fms-like tyrosine kinase 3 ligand; HLA-DR, human leukocyte antigen-DR; LPS, lipopolysaccharide; TNF-α, tumor necrosis factor alpha.

### 2.1 Monocyte and macrophages

Sepsis is characterized by a decrease in the capacity of monocytes from septic patients to release proinflammatory cytokines in response to endotoxins (lipopolysaccharide, LPS), other Toll-like receptor (TLR) agonists, and various bacterial compounds (41, 42). The activation of the adaptive immune system depends on monocytes and macrophages expressing the major histocompatibility antigen HLA-DR. One prevalent sign of immunosuppression in sepsis patients is decreased HLA-DR expression on monocytes. Numerous studies have shown that the expression of HLA-DR decreased on monocytes isolated from the bone marrow or peripheral blood of sepsis patients (43). This is correlated positively with nosocomial infection incidence and mortality (44, 45) and negatively with the clinical prognosis and sequential organ failure assessment (SOFA) score of sepsis (8, 46). Application of I interferons gamma (IFN-γ) to septic patients with low monocyte HLA-DR expression restored defects in HLA-DR expression and LPS-induced tumor necrosis factor alpha (TNF-α) secretion *in vitro*, which aided in the removal of pathogenic bacteria, according to a monocyte clinical trial that first reported this (29). Inhaled IFN-γ therapy can decrease the frequency of ventilator-associated pneumonia and restore the expression of HLA-DR in alveolar macrophages, without affecting the prognosis of patients with severe trauma (30). Treatment with IFN-γ increased mHLA-DR expression and restored TNF-α production in human endotoxemia, but further reduced the anti-inflammatory cytokine IL-10 production (31). A prospective, randomized, double-blind, placebo-controlled clinical trial discovered that septic patients receiving granulocyte-macrophage colony-stimulating factor (GM-CSF) had significantly higher monocyte mHLA-DR expression. These patients also experienced shorter hospital and intensive care unit stays, better acute physiology and Chronic Health Evaluation-II score, and shorter mechanical ventilation durations (25). There is considerable potential for treating sepsis immunosuppression through the phagocytosis and killing, antigen presentation, and secretion of pro-inflammatory cytokines by monocytes and macrophages. Thus, more investigation may yield novel ideas and strategies for the management of sepsis.

### 2.2 Neutrophils

Neutrophils are essential innate immune cells that possess a range of antimicrobial activities that enable them to ensnare and eliminate diverse pathogens, such as bacteria and fungi. Lower sepsis outcomes are linked to lower neutrophil counts, and the degree of immunosuppression caused by sepsis is correlated with lower bactericidal activity (47). According to the results of single-cell RNA sequencing, neutrophils underwent differentiation into multiple subclusters following LPS stimulation. Subsequent analysis revealed that LPS induced the overexpression of programmed death ligand 1 (PD-L1) on neutrophils via the p38α-MSK1/-MK2 pathway. In the direct contact mode, the subsets exhibiting elevated PD-L1 expression suppress the immune system by impeding T cell activation, inducing T cell apoptosis, and causing transdifferentiation (48). Septic neutrophils mediate lymphocyte apoptosis through a contact-dependent mechanism, which can be reversed by treatment with an anti-PD-L1 antibody (34). Subcutaneous GM-CSF (3μg/kg/day) administration for four days improved neutrophil phagocytosis and decreased the risk of secondary infection in sepsis patients, according to a phase IIa randomized clinical trial (32). Because neutrophils are crucial for the early invasion of pathogens, enhancing their function can enhance a number of biomarkers, lessen the severity of sepsis, and enhance the prognosis.

### 2.3 MDSCs

Myeloid-derived suppressor cells (MDSCs), a heterogeneous population of immature myeloid cells, are involved in immunosuppression in sepsis and are made up of both monocytic MDSCs and pathologically activated neutrophil MDSCs (49, 50). The transfer of normal neutrophils to suppressor cells in the bone marrow results in a major increase in MDSCs in immunosuppressed mice induced by LPS. In the bone marrow, MDSCs acquire suppressive activity and migrate to the lymph nodes to inhibit lymphocyte proliferation (51). The number of Gr1 (+) CD11b (+) MDSCs was markedly enhanced in a mouse model of polymicrobial sepsis caused by cecal ligation and puncture (CLP), and the number peaked during the late stage of sepsis (52). Sepsis survivors continued to have a significant proportion of MDSCs even after six weeks of infection control (53). Sepsis’ immunosuppressive condition should improve if the quantity of MDSCs is decreased or if their immunosuppressive effects are inhibited. According to research, gemcitabine has the potential to fully promote effector T cells during the gemcitabine cycle, decrease the amount of granulocyte MDSCs and Treg cells, and raise the ratio of effector T cells to Treg cells (35). By encouraging the death of spleen MDSCs, the liver X nuclear receptor (LXR) agonist GW396 can decrease the abundance of spleen MDSCs, improve bacterial clearance in tissues, and strengthen the immunosuppressive state of septic mice, all of which increase the survival rate of septic mice (36). According to recent research, the cyclooxy-genase-2 (COX-2) inhibitor NS398 can help the immunological disease that follows sepsis by preventing the growth and activity of MDSCs brought on by sepsis and boosting T-cell response (37). Given that sepsis causes long-term immunosuppression, MDSCs are thought to play a significant role in mediating immunological abnormalities in sepsis. Therefore, the phenotypes and biomarkers of MDSCs should be further studied for the target cells of immunosuppression.

### 2.4 NK cells

Natural Killer (NK) cells are pivotal in the realm of innate immunity, tasked with the recognition and elimination of pathogenic microorganisms, including viruses and bacteria. Despite their low representation in peripheral blood, studies have highlighted a substantial depletion of NK cell populations in sepsis patients, a phenomenon that has received limited attention in the literature (54). Nonetheless, NK cells are instrumental in the pathophysiology of sepsis. In sepsis patients, there is a marked upregulation of TLR2 and TLR4 expression in both the CD56^bright^ and CD56^dim^ NK cell subsets, concurrent with a reduced capacity to produce IFN-γ (55–58). Thymosin alpha1, a biologically active peptide synthesized by thymocytes, exerts immunomodulatory effects and is capable of modulating the activation of immune cells *in vivo*, including NK cells and monocytes (33, 38, 59). This property has been demonstrated to significantly reduce the mortality rate in sepsis patients (59). In conclusion, the restoration of NK cells function holds significant potential for the clinical management of immunosuppression induced by sepsis.

### 2.5 Dendritic cells

Dendritic cells (DCs), which are derived from bone marrow, serve as a pivotal component of the immune system by recognizing pathogens and are acknowledged as the most potent antigen-presenting cells. They act as a crucial bridge between the innate and adaptive immune responses, mediating immune tolerance. In the context of sepsis-related research, it has been observed that DCs are highly vulnerable to apoptosis triggered by sepsis (60), resulting in a reduction in both their quantity and functionality (11, 61–63). Several studies have demonstrated that the count of DCs in patients recovering from sepsis gradually normalizes several weeks post-sepsis (63), and treatment with Fms-like tyrosine kinase 3 ligand (FLT3L; also known as Dendritic cell growth factor) has been shown to significantly enhance the number and function of DCs, this, in turn, modulates the responsiveness of CD8 T cells to novel antigens (39). A study focusing on the CD155 receptor on DCs has indicated that blocking the overexpression of CD155 on DCs in sepsis can improve mouse survival rates by diminishing the bacterial load in both blood and peritoneal lavage fluid (40). Given the critical role of DCs in orchestrating the immune response, strategies to improve the quantity and functionality of DCs are of paramount importance for the amelioration of sepsis-induced immunosuppression.

## 3 Defining trained immunity

**Figure 2:**
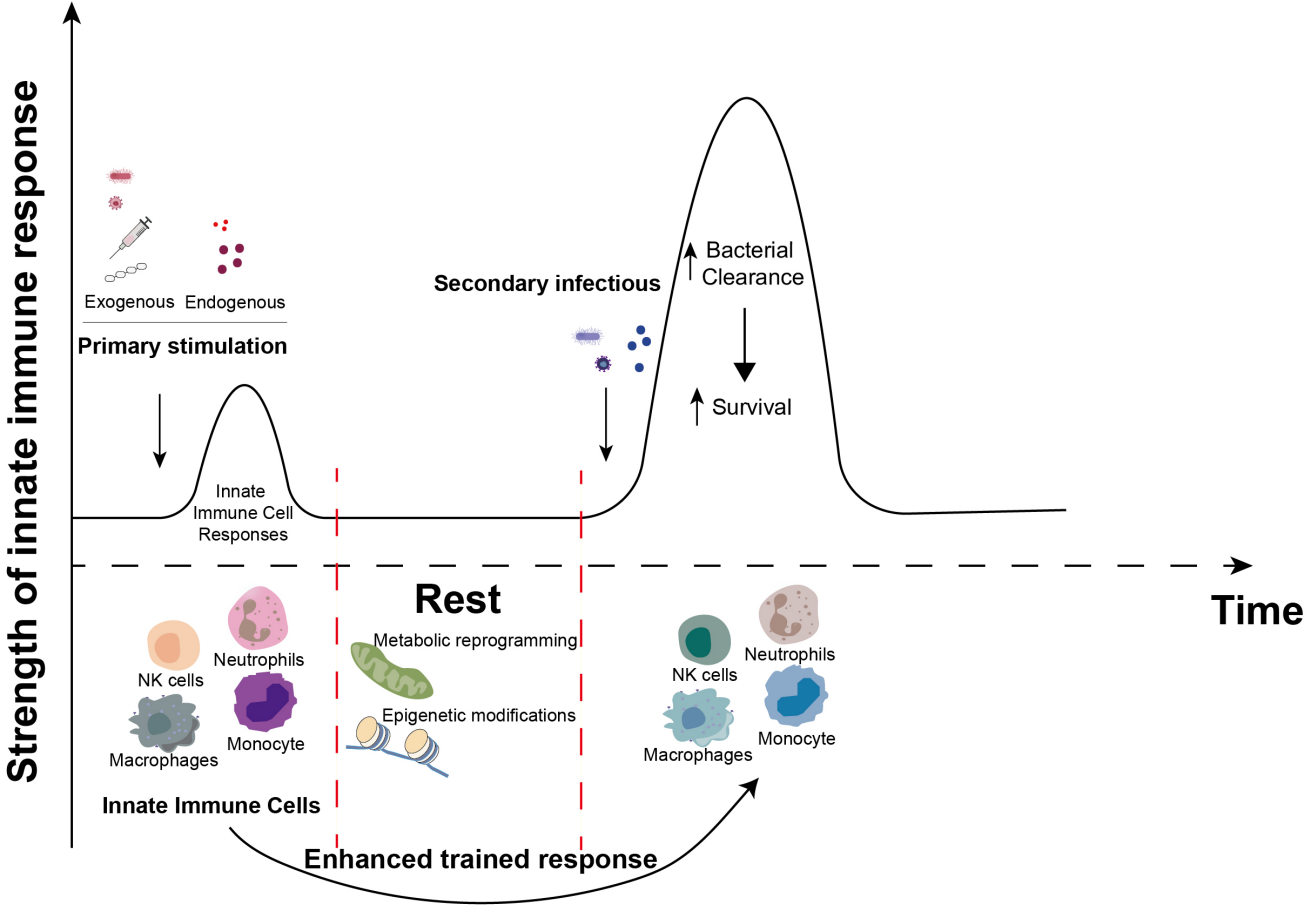
Induction of trained immunity. When innate immune cells (neutrophils, NK cells, monocytes and macrophages, etc.) are primarily stimulated by exogenous stimuli (bacteria, viruses, vaccines, β-glucans, etc.) or endogenous stimuli (heme, fumarate, etc.), complex metabolic reprogramming and epigenetic modification events occur during the corresponding immune response. These events persist in innate immune cells after the primary immune response is over. Upon subsequent exposure to certain stimuli, these previously activated innate immune cells elicit a more robust and accelerated response compared to the initial encounter, recruit additional immune cells, and enhance the clearance of invading microorganisms. This enhancement of innate antimicrobial efficacy due to the priming effect of prior exposure is termed “innate immune memory” or “trained immunity”.

In 2011, Professor Mihai Netea introduced the novel concept of trained immunity (64), which posits that innate immune cells possess a form of memory against pathogens they have previously encountered. This phenomenon, known as trained immunity (**Figure 2**), refers to the enhanced responsiveness of innate immune cells, such as monocytes/macrophages and NK cells, following the initial activation by a particular stimulus. Subsequent exposure to either the same or a different stimulus results in a more robust and expedited activation response compared to the initial activation.

Trained immunity signifies a long-lasting functional reset of innate immune cells, primarily driven by metabolic changes and epigenetic alterations (65). Various stimuli have the capacity to elicit distinct forms of trained immunity, meaning that the cells primed for trained immunity by different stimuli may differ or share similarities. β-glucan and Bacillus Calmette-Guerin (BCG) are currently the most commonly employed inducers for eliciting trained immunity. However, other factors such as heme, LPS and certain pathogens can also stimulate innate immune cells to develop trained immunity responses.

Research has indicated that the immunophenotype associated with trained immunity can persist for extended periods, ranging from 3 months to 1 year or more. Notably, a study examining the duration of nonspecific immunity induced by BCG vaccination in Uganda, Africa, found that the protection afforded by the attenuated BCG vaccine could last for as long as 5 years (18). In a double-blind, randomized Phase III trial, older adults who received the BCG vaccine exhibited a 79% reduction in infection rates compared to those who received a placebo one year following vaccination (66). Furthermore, a separate randomized Phase III trial demonstrated a 68% relative reduction in the risk of COVID-19 infection among older adults six months after BCG vaccination, as compared to those who received a placebo vaccine (67). Trained immunity has also been observed to influence cell differentiation and exhibit transgenerational effects. One study revealed that intravenous BCG vaccination in mice led to the expansion of hematopoietic stem and progenitor cells (HSPCs) and stimulated their differentiation into myeloid cells, thereby enhancing protection against Mycobacterium tuberculosis (MTB) infection (68). Additionally, recent investigations have shown that maternal-induced trained immunity can be transmitted to subsequent generations, underscoring the profound and enduring nature of this immune response (69, 70).

In the realm of immunology, it is widely understood that the adaptive immune system is the exclusive possessor of the attribute known as immune memory. This phenomenon is predominantly mediated by memory T and B lymphocytes, which are instrumental in safeguarding the host against subsequent infections. However, contemporary research has revealed that certain organisms, such as plants and invertebrates, which are devoid of an adaptive immune system, are capable of thwarting reinfection by pathogens, thereby exhibiting an enhanced host defense response. In the case of plants, this defense mechanism is composed of two main components: constitutive resistance, which is operative prior to the encounter with pathogens, and acquired resistance, which assumes paramount importance following pathogen detection. Researchers have termed the latter as “systemic acquired resistance (SAR)”, a process by which the plant can protect itself against a multitude of pathogens (71–74). Invertebrates, lacking functionally equivalent cells to T and B lymphocytes, do not engage in adaptive immune responses. Nevertheless, this does not preclude the existence of immune memory in these organisms. Numerous studies have elucidated the presence of trained immunity characteristics in invertebrates, suggesting that insects, once infected with Streptococcus pneumoniae or Beauveria bassiana, are shielded from reinfection with the same pathogen but remain susceptible to other pathogens (75). This trained immunity has also been evidenced in other invertebrates, including the malaria vector Anopheles gambiae (76), crustacean copepods (77), and shrimp (78). The concept of trained immunity represents a novel extension of immunological principles. It not only offers a fresh perspective for investigating immunological mechanisms but also introduces a novel therapeutic strategy for the treatment of various diseases.

## 4 Activating trained immunities to fight sepsis

**Figure 3:**
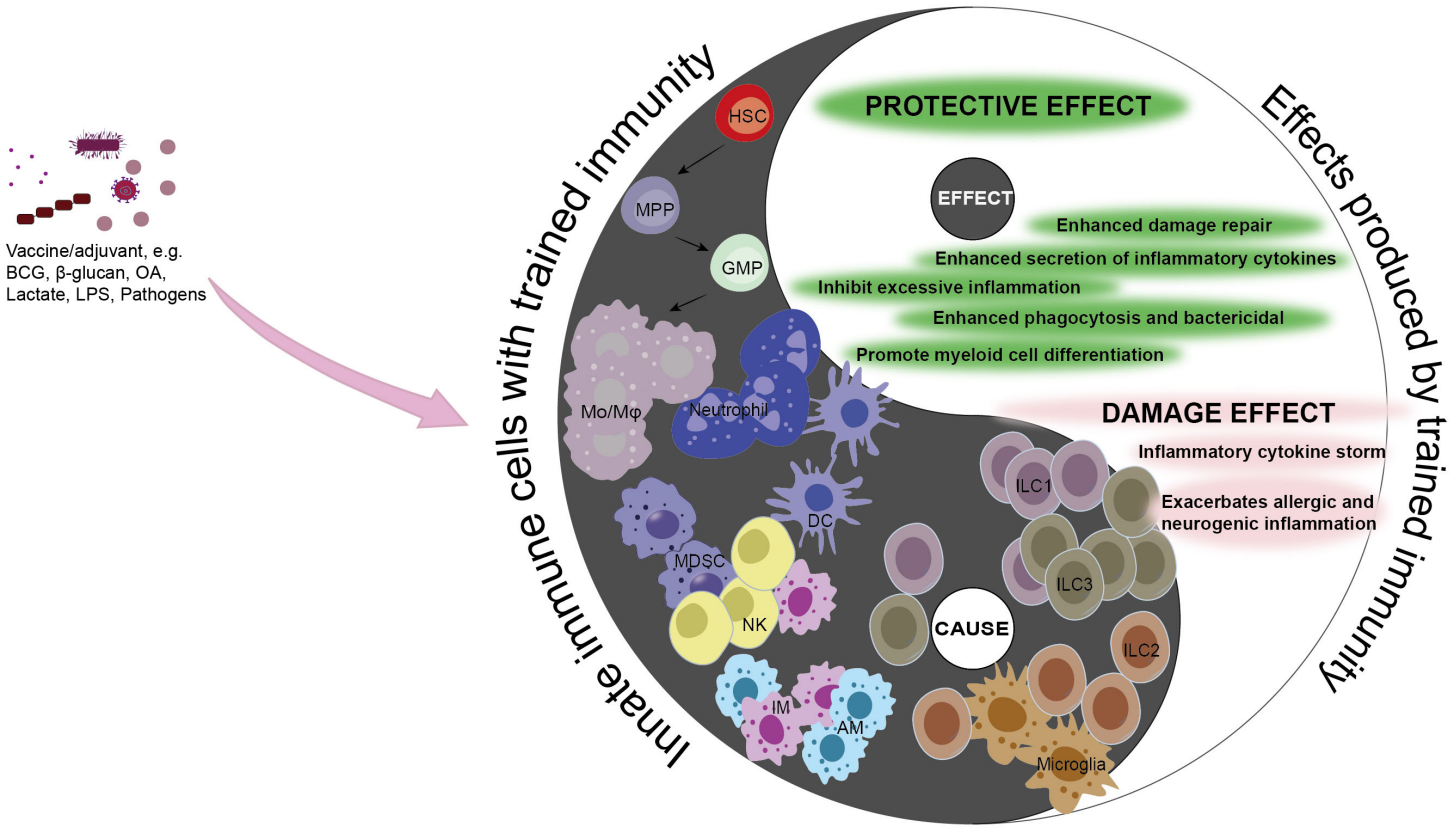
Causality of trained immunity in sepsis. Trained immunity is a protective mechanism aimed at shielding the host from infection and reinfection. However, the adverse effects of trained immunity can lead to excessive inflammation, immune tolerance, or immunosuppression, thereby disrupting the balance of the immune system. The double-edged sword effect of trained immunity is consistent with the causal relationship in traditional Chinese philosophy, where the occurrence of trained immunity in innate immune cells is the “cause”, and the impact it has on the disease or the host is the “effect”. Under the action of corresponding stimuli, differentiate hematopoietic stem cells into myeloid cells, enhancing the capacity to clear pathogens and strengthening the killing functions of innate immune cells such as Mo/Mφ, NK cells, ILC1s, ILC3s, AMs, IMs, and DCs, thereby promoting host protection. MDSCs can inhibit the excessive inflammatory response caused by pathogens and mitigate the damage to the host. Conversely, neutrophils, ILC2s, and microglia can trigger excessive inflammation, and excessive stimulation can lead monocytes to an immunosuppressed state, harming the host. Black, the Innate immune cells with trained immunity (cause); white, the effects produced by Innate immune cells with trained immunity (effect). Mo/Mφ, monocyte/macrophage; HSC, hematopoietic stem cell; MPP, multipotent progenitor; GMP, granulocyte-macrophage progenitor; MDSC, myeloid-derived suppressor cell; NK, natural killer cell; AM, alveolar macrophage; IM, lung interstitial macrophage; DC, dendritic cell; ILC1, Group 1 innate lymphoid cell; ILC2, Group 2 innate lymphoid cell; ILC3, Group 3 innate lymphoid cell; BCG, Baccillus Calmette-Guerin; LPS, lipopolysaccharide; OA, oroxylin A.

Throughout the past century, infectious diseases have remained one of the leading causes of mortality globally, and to this day, low-income countries continue to grapple with this significant health challenge. Sepsis, in particular, can lead to severe organ damage or death due to immunosuppression or an excessive inflammatory response. Consequently, there is a critical need for therapeutic strategies that can restore balance to the immune response in sepsis patients and enhance their outcomes through immunotherapeutic interventions. In the last few decades, advancements in immunological research have uncovered the concept of trained immunity. This is a state wherein innate immune cells are primed through epigenetic and metabolic reprogramming, thereby providing more robust protection against subsequent infections (**Figure 3**). This innovative understanding of the immune response has the potential to significantly improve survival rates in sepsis patients.

### 4.1 Monocytes and macrophages

Recent Single-cell RNA sequencing studies have revealed that the trained immune subsets of monocytes are suppressed in patients with severe sepsis. This suppression may compromise the pathogen-phagocytosing and clearing capabilities of monocytes, potentially exacerbating the severity of the disease (79). Consequently, there is an imperative need to enhance the proportion and functional integrity of immuno-trained monocytes in sepsis patients. Such improvements could markedly elevate the survival rates among these patients.

Recent scholarly endeavors have underscored the immense potential of monocytes from septic patients to contribute to the treatment of sepsis following a period of training. Notably, when primed with β-glucan, these monocytes exhibit enhanced metabolic capacity and cytokine secretion, alongside a substantial increase in both cellular respiration and glycolysis rates (80–82). β-glucan’s ability to effectively rewire macrophages has also been observed, fostering the production of trained immune markers and bolstering bacterial phagocytosis, thereby offering protection against sepsis and secondary infections mediated by pathogenic bacteria and CLP (83–85). Furthermore, research has uncovered that interleukin-4 (IL-4) not only suppresses acute inflammation but also instills long-term innate immune memory in monocytes. This is achieved through the upregulation of anti-inflammatory factors, such as IL-1Ra, and the modulation of genes associated with trained immunity, including proinflammatory cytokines like IL-6 and IL-12B, while concurrently downregulating immunosuppressive genes like Chemokine (C-C motif) ligand 19 (CCL19) and Suppressor of cytokine signaling 2 (SOCS2) (86). The tuberculosis vaccine, BCG has demonstrated a diverse and enduring beneficial effect against unrelated infections. In a randomized placebo-controlled human challenge study, BCG vaccination was found to elicit genome-wide epigenetic reprogramming of monocytes, resulting in a significant augmentation of inflammatory cytokine production, including TNF-α, IL-1β, and IL-6, by peripheral blood monocytes in response to nonspecific stimulation in volunteers within the BCG cohort, as compared to the placebo group. This intervention also mitigated viremia following yellow fever virus vaccination and maintained protection for at least one month (87). Heme, characterized as a damage-associated molecular pattern (DAMP) (88, 89) or alarmin (90), has been recognized for its capacity to elicit inflammatory responses through pattern recognition receptors (PRRS) (91). A study focusing on heme-induced trained immunity has demonstrated that heme can activate spleen tyrosine kinase (SYK) and c-Jun N-terminal kinase (c-JNK) signaling pathways in human monocytes and macrophages, thereby inducing trained immunity. This process enhances the expression of myeloid differentiation-related genes, such as Nfix and Runx1, and the secretion of cytokine TNF, thereby bolstering resistance to pathogens and improving the survival rate of sepsis-afflicted mice (92). Moreover, the utility of employing a combination of inducers for trained immunity has been explored. For instance, the synergistic use of BCG and bacterial lipoprotein (BLP) has been shown to induce trained immunity in neonatal mouse macrophages, thereby enhancing their inflammatory response, antibacterial capabilities, and the production of inflammatory cytokines and chemokines, as well as potentiating bactericidal activity (93). Certain components of traditional Chinese medicine have been shown to elicit trained immunity. Oroxylin A (OA) has been identified as a compound that mitigates sepsis by fostering the trained immune response in macrophages and enhancing LC3-associated phagocytosis (LAP). Additionally, OA has been demonstrated to regulate LAP via the Dectin-1-Syk axis, thereby augmenting the pathogen-elimination capacity of macrophages and effectively alleviate sepsis (94).

In an era marked by a critical shortage of medical resources and the persistent challenge of pathogenic infections, the body’s innate immune cells engage in a process of long-term genetic reprogramming to mount effective defenses against the invasions and infections of diverse pathogenic microorganisms. Sepsis results in enduring and profound modifications to the immune system for an extended period, typically several months (95). These alterations are characterized by the enhancement of immune tolerance or the development of trained immunity (96–98). Extensive research on the induction of trained immunity by microbial infections has demonstrated that animals, such as mice, can elicit a trained immune response in their macrophages or monocytes following initial infection with pathogenic microorganisms. Confronted with subsequent infections, whether they be homologous or heterologous to the initial pathogen, these animals exhibit heightened immune responses, increased cytokine production, enhanced pathogen lethality, and improved survival rates (83, 99–101). For instance, intraperitoneal injection of inactivated Candida albicans has been shown to induce bone marrow-derived macrophage (BMDM) trained immunity in mice, which augments their capacity to clear pathogens and promotes the release of extracellular traps (ETs). This intervention significantly enhances the resistance of mice to subsequent infections by Clostridium perfringens (102). Studies exploring the dosage effects of LPS have revealed that the immune-modulating impact of LPS dosage varies. High doses of LPS have been found to induce macrophage immune tolerance (82, 103, 104), whereas low doses of LPS can effectively prime macrophages to develop trained immunity responses( (105, 106). A recent study has revealed that repetitive high-dose LPS stimulation can drive monocytes towards an exhausted phenotype. Paradoxically, the generation of immune memory cells also hinges on continuous stimulation, yet the outcomes are antithetical. Exhausted monocytes are distinguished by upregulated expression of pro-inflammatory and immunosuppressive genes, coupled with downregulated expression of costimulatory molecules. This is exemplified by an increase in Ly6C^hi^ monocytes and a decrease in Ly6C^lo^ monocytes, mitochondrial dysfunction, and elevated levels of reactive oxygen species (ROS) (107). Concurrently, extensive DNA methylation changes were observed in the genome of LPS-stimulated exhausted monocytes, predominantly within enhancers and promoters, and these alterations were associated with modified expression of genes involved in immune dysregulation. Furthermore, similar DNA methylation pattern changes were detected in bone marrow monocytes from septic mice, resembling the modifications observed *in vitro* in exhausted monocytes cultured. These findings suggest that DNA methylation plays a pivotal role in the establishment and maintenance of the exhausted monocyte phenotype during sepsis (108). Exhausted monocytes contribute to immunosuppression through the upregulation of immunosuppressive molecules, alterations in quantity, and dysfunction, potentially representing a significant mechanism underlying the chronic immune suppression observed in patients with sepsis. These findings underscore the intricacies of the immune response to microbial challenges and suggest that the modulation of immune cell programming may represent a strategic avenue for the enhancement of host defenses against infectious diseases, particularly in resource-constrained environments.

Pathogens, such as bacteria and viruses, are key triggers for the pathogenesis of sepsis, with the severe acute respiratory syndrome coronavirus 2 (SARS-CoV-2) responsible for the COVID-19 pandemic (109). Research has revealed that patients with acute COVID-19 exhibit a reduction in the total number of monocytes and a decrease in the expression of HLA-DR, indicating a state of immune dysregulation. However, there is increased chromatin accessibility observed in the monocytes of patients with acute COVID-19, and certain transcriptomic and epigenetic features persist even six months after recovery. These findings suggest that COVID-19 infection may result in enduring epigenetic modifications in monocytes, which could potentially contribute to the formation of immunological memory (110). A recent study has indicated that months to a year following recovery from COVID-19, monocytes exhibit epigenetic and transcriptomic persistence, resulting in an overly activated state with enhanced inflammatory responsiveness, migration, and differentiation capabilities. These persistent epigenetic alterations may underpin the persistence of monocytes post-COVID-19 infection and their engagement in chronic inflammatory responses (111).

Trained immunity, as an emerging immunotherapeutic strategy, has garnered significant interest in the field of immunology. However, sepsis, a complex clinical condition, frequently results in disease exacerbation and poor patient outcomes, partly due to the immunosuppressive state it induces. It has been well-documented that the immunosuppression observed in sepsis patients is associated with elevated serum lactate levels, and approximately two-thirds of patients with severe sepsis or septic shock commonly present with hyperlactatemia. Recent scholarly contributions have elucidated that lactate exerts regulatory influence over cellular metabolism and that the acetylation of histone H3 lysine 27 (H3K27ac) elevates the expression of the nuclear receptor subfamily 4, group A, member 1 (Nr4a1), thereby inhibiting the pro-inflammatory response induced by LPS in macrophages. This modulation downregulates the expression and production of pro-inflammatory factors. Furthermore, lactate preconditioning has been shown to instigate a persistent immunosuppressive state within macrophages, persisting even after subsequent LPS exposure, leading to a significantly attenuated inflammatory response. This phenomenon, termed “trained immunosuppression”, highlights the complexity of the immune response to sepsis (112). In light of these findings, the manipulation of lactate production or enhancement of lactate clearance emerges as a potentially crucial strategy to counteract the immunosuppressive and detrimental effects of lactate in septic patients. Inhibiting lactate production or increasing its clearance may be as vital as promoting trained immunity itself, in the context of improving outcomes for patients suffering from sepsis.

### 4.2 Neutrophils

Neutrophils, as the most prevalent type of white blood cells in the circulation, serve as the vanguard of the innate immune system’s response to infections (113). These cells are summoned from the bone marrow to the peripheral bloodstream, where they engage in phagocytosis to eliminate pathogens during the course of infection or inflammation (114). However, in the context of sepsis, neutrophils exhibit delayed apoptosis, a phenomenon that contributes to the persistence of inflammation and can lead to long-term consequences (115). A pivotal study has revealed that sepsis-induced neutrophils are capable of fostering trained immunity, characterized by enhanced glycolysis, fatty acid synthesis, and epigenetic modifications. These changes manifest as an amplified production of TNF-α, a heightened respiratory burst, and a significantly improved capacity for phagocytosis when the neutrophils encounter a secondary challenge, such as exposure to LPS or a bacterial infection. While trained neutrophils exhibit enhanced pathogen-clearance abilities, they also have a propensity to migrate to the lungs, which can exacerbate the extent of acute lung injury (116). The excessive inflammatory response triggered by these immune-trained neutrophils has the potential to cause damage to the host, particularly in relation to tissue injury and organ failure. Therefore, the modulation of neutrophil-mediated trained immunity assumes paramount importance in the management of sepsis. By regulating the trained immunity of neutrophils, it may be possible to mitigate the inflammatory surge attendant to secondary infections, thereby enhancing the prognosis of patients with sepsis. Neutrophils are also capable of manifesting an exhausted phenotype. Neutrophils that are depleted exhibit increased expression of CD11b, lose their capacity for an effective inflammatory response, demonstrate reduced expression of inflammatory mediators, and exhibit disrupted cell metabolism along with elevated elastase secretion, which can lead to tissue damage (117). Conversely, neutrophils with Toll/IL-1 receptor domain-containing adaptor molecule (TRAM) deletion exhibit “memory” properties that enable them to maintain a state of reduced inflammation and secrete greater amounts of anti-inflammatory factors, thereby preventing the development of an exhausted state. This “memory” attribute may play a significant role in the immunomodulatory function during sepsis (117).

### 4.3 MDSCs

MDSCs constitute a heterogeneous cell population that serves a critical role in modulating both innate and adaptive immune responses, particularly those involving T cells, and have become an integral component of the immune response to sepsis. Gr-1+ granulocyte MDSCs, which function as effector cells capable of eliciting trained immunity, can be generated in the bone marrow through intraperitoneal immunization with low-virulence strains of Candida, such as Candida dubliniensis and Candida glabrata. Upon exposure to a lethal intra-abdominal infection involving Candida and Staphylococcus aureus, these MDSCs have been shown to mitigate the excessive inflammatory response that follows, thus safeguarding mice from fatal sepsis (100, 101, 118–120). The mechanism of protection conferred by this trained immunity is strain-dependent, with less virulent Candida strains eliciting a more robust protective response (118). Importantly, protection induced by Candida dubliniensis does not necessitate hyphal formation, and innate immune memory can be established as early as day 7 post-primary infection, persisting beyond 60 days (101, 120). The immune training mediated by hypovirulent fungi holds significant promise for the development of novel vaccine strategies. With a deeper understanding of the cellular and molecular underpinnings of this process, it is increasingly evident that MDSCs represent a promising therapeutic target for the clinical management of sepsis-associated immunosuppression.

### 4.4 NK cells

NK cells serve as effectors within the innate immune system, being present both in the peripheral blood and within various tissues. They are pivotal in the defense against infections and tumors, as well as in the modulation of the immune response (121–124). Established research has demonstrated that NK cells are capable of undergoing clonal expansion, epigenetic reprogramming, and the differentiation into long-lived populations (121, 125, 126). Recent investigations have further established that NK cells possess characteristics akin to immune memory. Specifically, viral and bacterial skin infections can elicit the recruitment of circulating conventional NK (cNK) cells and the differentiation of Tcf1^hi^ CD69^hi^ tissue-resident NK (trNK) cells, facilitating a rapid and enhanced response to subsequent infections (127). Despite these advances, there remains a substantial gap in our understanding of NK cells in the context of sepsis treatment. Consequently, a deeper exploration of the immune memory functions of NK cells is imperative and is expected to yield novel insights and strategies for the management of sepsis.

### 4.5 HSCs

It is widely acknowledged that the primary targets of trained immunity are innate immune cells. However, research has uncovered that trained immunity also manifestations in central immune organs, such as the bone marrow. Hematopoietic stem cells (HSCs) are unique within the human blood system, possessing self-renewal capabilities and multipotent differentiation potential. These cells are capable of generating hematopoietic progenitor cells with diverse lineage potential (128). Hematopoietic stem cells within the bone marrow of mice can be activated by heme to foster immunity, facilitating the differentiation of these stem cells along the myeloid lineage and increasing the frequency of myeloid progenitor cells. This activation reduces the *in vivo* pathogen load and enhances the resistance of mice to bacterial sepsis, with this protection enduring for at least 28 days (92). The immune-training inducing agonist β-glucan has been shown to elicit trained immunity in HSPCs within the bone marrow. This process helps safeguard hematopoietic progenitor cells from DNA damage, increases the proportion of myeloid progenitor cells, and ameliorates the response to secondary inflammatory stimuli and chemotherapy-induced myelosuppression (129). The cross-protective efficacy of BCG and its capacity to train monocyte-macrophage (Mo/Mac) cells *in vitro* have been well-documented in numerous studies (130–133). Intravenous BCG administration influences the transcriptome of HSCs and multipotent progenitor cells, thereby promoting the differentiation of granulocyte-lineage progenitors while suppressing the differentiation of lymphocyte-lineage progenitors. This results in the generation of epigenetically modified macrophages, which can markedly enhance protection against Mycobacterium tuberculosis infection (68). Research has revealed that patients who recover from severe COVID-19 continue to exhibit enduring epigenetic and transcriptomic alterations in HSPCs for up to one year post-infection. These alterations, which are predominantly linked to epigenetic modifications of inflammatory genes, result in HSPCs that are super-reactive to stimuli. The epigenetic “memory” of HSPCs can be heritable by their progeny, resulting in monocytes that display similar persistent epigenetic and transcriptomic modifications. This inheritance of epigenetic alterations may contribute to the long-term immune consequences of severe COVID-19 infection (111). Trained immunity thus impacts not only mature myeloid cells but also exists within the bone marrow milieu. Although hematopoietic stem cells are the primary effector cells, they ultimately differentiate into myeloid cells and bolster the response to inflammatory triggers.

### 4.6 ILCs

Innate lymphoid cells (ILCs) are a class of tissue-resident lymphocytes that arise from a common lymphoid progenitor (CLP), which differentiates into precursors committed to specific cell lineages (134–136). As one of the latest additions to the family of innate immune cells (137, 138), ILCs can be primed to develop trained immunity (139–141). Distinct from T and B cells, ILCs do not bear diverse antigen receptors; instead, they express a array of germline-encoded activating and inhibitory receptors and are capable of producing various effector cytokines (142, 143). ILCs operate through cell-surface molecular interactions and cell-cell interactions, playing a critical role in the early defense of the host against pathogens at the site of initial infection. This is particularly crucial for protecting epithelial barriers from infection and for maintaining organ homeostasis. Mature ILCs exhibit considerable phenotypic and functional heterogeneity among their members and can be generally categorized into three major groups based on the co-expression of surface markers, transcription factors, and effector cytokines that define ILCs (144–147).

#### 4.6.1 ILC1s

Group 1 innate lymphoid cells (ILC1s) are characterized by their rapid response following infection (148). They respond to the pro-inflammatory cytokine IL-12 and express the transcription factor T-bet (149, 150), generating a range of effector cytokines and cytotoxic mediators, including IFN-γ, TNF-α, and granzyme-containing cytotoxic granules. This response functions to protect the host from bacterial and viral pathogens at the site of initial infection (134, 148, 151). A study has demonstrated that liver-resident type 1 ILCs (ILC1s) locally expand and persist following the resolution of mouse cytomegalovirus (MCMV) infection. They establish memory responses in an antigen-dependent manner, characterized by stable transcriptional, epigenetic, and phenotypic alterations. These cells also exhibit enhanced protective effects upon re-challenge with MCMV (139).

#### 4.6.2 ILC2s

Group 2 innate lymphoid cells (ILC2s) are activated in response to cytokines such as IL-25, IL-33, and TSLP. Under the regulation of the transcription factor GATA-3 and the orphan receptor RORα (152, 153), ILC2s orchestrate innate type 2 immune responses against various challenges, including allergen exposure, parasitic infections, and respiratory virus infections. This is achieved through the production of TH2 cytokines (IL-4, IL-5, IL-9, IL-13, etc.) and amphiregulin (AREG) upon cell activation (152, 154–156). Research has indicated that ILC2s exposed to allergens acquire memory-like properties and exhibit a heightened response to challenge with unrelated allergens compared to non-exposed ILC2s, potentially exacerbating hypersensitivity pneumonitis (140).

#### 4.6.3 ILC3s

Group 3 innate lymphoid cells (ILC3s) are highly enriched in the intestinal tract and play a pivotal role in antibacterial immunity (157, 158). These cells are crucial effector cells at mucosal barriers, coordinating the development of lymphoid tissue and mucosal defense (159–161). ILC3s respond to cytokines such as IL-1β, IL-6, and IL-23, express RORγt constitutively (162), and produce IL-17 and IL-22. These cytokines promote the production of antimicrobial peptides by intestinal epithelial cells, thereby establishing immune memory against pathogens (157, 158, 162–165). Following exposure to Citrobacter rodentium, intestinal ILC3s remain activated and undergo metabolic changes for several months. This results in enhanced proliferative capacity and an enhanced response to IL-22 during reinfection, leading to improved control of the infection (141).

ILCs, as resident immune cells (166), are capable of eliciting protective immunity in response to infection. They facilitate wound healing and mitigate tissue damage, contributing to the maintenance of organizational integrity. The cytokines produced by ILCs are instrumental in effectively eliminating pathogens and maintaining immune homeostasis (167–169). Extensive research has underscored the increasingly critical role ILCs play in the immune response during sepsis (170–172). Consequently, inducing a trained immunity state in ILCs is anticipated to enhance the immune response within mucosal tissues, facilitate more effective pathogen clearance, and improve the prognosis of sepsis patients.

### 4.7 Resident lung tissue macrophages

Alveolar macrophages (AMs) and lung interstitial macrophages (IMs) are abundant within the lung tissue, constituting the predominant resident macrophages that together form the core of the lung’s innate immune system. These cells are characterized by their robust phagocytic, immune, and secretory capabilities, which enable them to effectively clear inhaled dust particles, bacteria, and other airborne foreign matter. They fulfill essential homeostatic, metabolic, and repair functions within the lung, thereby playing a critical role in the lung’s defense mechanism against infections and maintaining its overall health and functionality (173).

#### 4.7.1 AMs

AMs are an abundant subset of innate immune cells within the lung, serving as long-lived, tissue-resident macrophages. These cells act as sentinels, primarily responsible for the recognition and clearance of pathogens or pollutants within the lung, and are pivotal in the innate immune response of the respiratory tract (174, 175). Recent research has shown that subcutaneous BCG vaccination can trigger the generation of memory alveolar macrophages and trained immunity through the gut-lung axis (176). This process is independent of circulating monocytes and is mediated by the transmission of mycobacteria, which leads to temporal changes in the gut microbiome, gut barrier function, and microbial metabolites. These changes facilitate the survival of macrophages in the lungs and the induction of trained immunity, which is beneficial for the early control of tuberculosis mycobacterium infections and the reduction of lung infection severity (176). Respiratory viral infections can also elicit long-lasting memory in alveolar macrophages. During the initial stages of viral infection, AMs require the priming assistance of CD8 T cells, but once memory AMs are formed, they can maintain their functionality independently. Trained alveolar macrophages are capable of enhancing host defense by promptly producing chemokines and increasing neutrophil infiltration to bolster resistance against bacterial infections (177). Remarkably, influenza virus-trained AMs have been shown to induce anti-tumor immunity in the lung. These trained AMs are able to infiltrate tumor tissue and enhance their phagocytic and cytolytic capabilities against tumor cells, while also countering the immunosuppressive effects of the tumor microenvironment (178). Repeated exposure to pathogens or pathogen components can stimulate and train AMs to augment host defense responses. Furthermore, recent studies have indicated that the immune memory and repair capacity of AMs are altered after repeated challenges with bacterial endotoxin or Pseudomonas aeruginosa (179). Training can induce the expansion of the MERTK^hi^ Marco^hi^ CD163^+^ F4/80^low^ subset of lung resident AMs, which exhibit a proresolving phenotype, greater resistance to pathogen-induced cell death, and enhanced capacity for phagocytosis of debris and repair of damage, thereby limiting inflammatory lung injury (179). There is evidence of a certain interconnectedness between different diseases, which may involve mutual promotion or inhibition. Sepsis and tumor are both highly detrimental to the human body, yet their relationship is complex. Survivors of sepsis have been found to have a significantly reduced risk of developing cancer. Following sepsis resolution, sepsis-induced trained immunity in AMs undergoes epigenetic changes, endowing them with innate immune memory. This is associated with an increased production of chemokines such as CXCL16, which promotes the tissue residency of CXCR6^+^ T cells and reduces the risk of *de novo* tumor formation (180).

#### 4.7.2 IMs

IMs serve as vital defenders of the vasculature and lung interstitium (181), possessing a crucial immune function that is essential for maintaining lung homeostasis and preventing immune-mediated allergic airway inflammation. A recent investigation has elucidated that the trained immunity elicited by whole β-glucan particles (WGP) is orchestrated by the metabolite sphingosine 1-phosphate. Furthermore, the activated lung interstitial macrophages exhibit antitumor capabilities, which effectively inhibits diverse metastatic processes in mice and augments survival time during the onset of tumor metastasis (182). The phagocytic capacity, cytotoxicity, metabolic reprogramming, and T cell regulatory functions of IMs following WGP-induced training are significantly amplified. These findings suggest that trained IMs may hold profound implications in the therapeutic management of sepsis-induced lung injury, with the potential to enhance patient prognosis.

The lung is a critical organ significantly affected by sepsis, and lung tissue-resident macrophages represent essential immune cells tasked with preserving the equilibrium of the lung’s internal milieu. The evocation of trained immunity within these macrophages may represent a promising anti-inflammatory and anti-cancer approach, holding the potential to introduce novel therapeutic avenues for mitigating or remedying sepsis-induced lung injury in clinical settings.

### 4.8 Microglias

Microglia are brain-resident macrophages that are diffusely distributed throughout the central nervous system (CNS) parenchyma (183). As exceedingly long-lived cells, they establish a cellular network through ongoing processes of branching and migration. They play a pivotal role in neural circuit remodeling and synaptic function, which is vital for the optimal operation of the brain (184–187). Additionally, they are intimately involved in the pathogenesis of nervous system diseases. The research revealed that peripheral inflammation can precipitate acute immune training and tolerance in microglial cells within the brain, resulting in distinct epigenetic reprogramming that persists for at least six months. In an Alzheimer’s disease mouse model, immune training exacerbates β-amyloid deposition, while tolerance attenuates it (188). Neonatal infection can induce trained immunity in microglia, leading to compromised function and an overactive response to low doses of amyloid-β oligomers (AβOs), thereby triggering increased neuroinflammation, synaptic loss, and cognitive impairment (189). The detrimental effects of trained microglia in the CNS underscore the significance of immune memory as a regulatory factor, offering novel insights into the pathology of neurodegenerative diseases and sepsis-induced brain dysfunction.

### 4.9 Dendritic cells

DCs are a pivotal component of the innate immune system. Historically, DCs have been perceived as lacking antigen specificity and immune memory; however, they are indispensable for the initiation and modulation of immune responses. Contemporary research has delved into the observation that DCs exhibit characteristics akin to memory cells. For instance, Cryptococcus neoformans has been demonstrated to trigger the development of trained immunity in DCs (190). DCs isolated from mice exposed to Cryptococcus neoformans have been shown to enhance their capacity to produce IFN-γ and other pro-inflammatory cytokines in response to subsequent pathogenic challenges, thereby improving pathogen clearance (190). By manipulating the polarization and immune memory potential of DCs, the host’s immune response against specific pathogens can be potentiated, thereby offering a preventive and therapeutic advantage against diseases. This discovery also presents a novel perspective for the clinical management of sepsis.

## 5 Mechanism of trained immunity in sepsis

As medical science continues to advance, the role of immunosuppression in sepsis has come to light as a critical factor influencing patient survival rates and outcomes. Dysfunction and depletion of immune cells are primary triggers of immunosuppression. Trained immunity is a phenomenon that is coordinately regulated by both long-term metabolic reprogramming and histone epigenetic modifications, thereby altering the long-term responsiveness of innate immune cells. This conditioning enables the immune cells to mount a more rapid and robust response upon re-exposure to either homologous or heterologous stimuli, thereby enhancing the host’s immune defense mechanisms. In the following section, we will provide a concise overview of the mechanism underlying the induction of trained immunity in the context of sepsis.

In quiescent myeloid cells, the majority of genes encoding for inflammation are silenced in a repressive chromatin state (191–193). Upon initial stimulation, there is a dynamic transformation at these loci, characterized by increased chromatin accessibility and altered histone modifications, including H3K27ac, H3K4me1, and H3K4me3. These epigenetic modifications facilitate the recruitment of transcription factors to the enhancer or promoter regions, leading to the rapid transcription of inflammatory genes, such as TNF-α and IL-6. Studies have demonstrated that β-glucan activates the Dectin-1 receptor, triggering a cascade of downstream signaling events that include the activation of Protein Kinase B (Akt) and Mammalian Target of Rapamycin (mTOR) (80). β-glucan inhibits the expression of LPS-induced immune response gene 1 (IRG1) and preserves the function of succinate dehydrogenase (SDH) through the IRG1/itaconate/SDH axis (81). Additionally, β-glucan regulates glucose metabolism by shifting from oxidative phosphorylation to aerobic glycolysis, via the AKT/mTOR/Hypoxia-Inducible Factor 1α (HIF-1α) pathway, which results in increased lactate production (194), modulated cellular metabolism, and the induction of trained immunity. Furthermore, the trained immunity elicited by β-glucan has been shown to reverse LPS-induced tolerance by enhancing H3K27ac and H3K4me1 at the promoter and enhancer regions of genes involved in lipid metabolism and phagocytosis, thereby restoring the levels of inflammatory cytokines in LPS-treated monocytes (82). The BCG vaccine has been found to activate the mTOR signaling pathway, suppress lymphocyte-related pathways, induce epigenetic reprogramming (86), and induce genome-wide modifications in the histone H3K27ac mark in human monocytes, regulating the gene promoter regions of pro-inflammatory cytokines such as TNF-α and IL-6 (87). Candida albicans mediates an increase in H3K4me3 through the Dectin-1/Raf-1 pathway, leading to epigenetic and transcriptomic changes in monocytes and enhancing their immune response (83). Upon LPS stimulation, the formation of exhausted monocytes is accompanied by extensive DNA methylation alterations across their genome, concomitant with diminished levels of H3K27ac in enhancer regions (108). The TIR domain-containing adapter molecule 2 (TICAM2) signaling pathway modulates the extent of DNA methylation by governing the activity of transcription factors PU.1 and IRF8. TICAM2 emerges as the principal upstream regulator of the TRAM signaling cascade. Knockout of the TICAM2 gene was found to partially counteract the LPS-induced phenotype of monocyte exhaustion, including the associated changes in DNA methylation (108). Furthermore, depletion of TRAM similarly inhibited LPS-induced monocyte exhaustion, curtailed the expansion of Ly6C^hi^ monocytes, reduced the expression of CD38 and PD-L1, and partially reinstated CD86 expression (195). These findings suggest that TRAM signaling may be crucial for the initiation of DNA methylation processes.

While we have touched upon the mechanism of training immunity induction in sepsis previously, our discourse has not delved into the intricacies of this process. The development of trained immunity is a multifactorial outcome, and there is a wealth of evidence suggesting that it is also intertwined with cellular metabolism. Alterations in metabolic pathways regulate gene transcription and play a pivotal role in the establishment of trained immunity. In the context of sepsis, there are pronounced metabolic shifts within various cell types. However, whether manipulating these metabolic pathways can be leveraged to foster training immunity and thereby enhance sepsis outcomes remains an area ripe for exploration.

## 6 Discussion

Sepsis is a systemic inflammatory response syndrome that arises from a pathogenic infection, and its development is intricately linked to the dysregulated and excessive activation of the immune system (196). In the setting of sepsis, the immune system’s hyperresponse leads to the massive release of inflammatory mediators, which, in turn, ignites a cascade of inflammatory reactions that inflicts damage upon normal tissues and organs. Concurrently, the regulatory capacity of the immune system is compromised, resulting in immunosuppression that diminishes the body’s capacity to eliminate pathogens, rendering the patient more susceptible to secondary infections. This exacerbates the disease’s severity and increases the mortality rate among patients.

Trained immunity, as an evolving immunomodulatory approach, warrants significant recognition in the realm of immunotherapy. Its potential to enhance the efficacy of immunotherapeutic interventions and to revolutionize the treatment landscape for diverse diseases should not be trivially dismissed. By judiciously activating the immune system, trained immunity can prompt immune cells to manifest a memory-like response. This mechanism holds tremendous promise in refining the precision of immunotherapy targeting, circumventing drug resistance issues, augmenting the spectrum of immune response, restoring immune homeostasis, and mitigating adverse reactions associated with immunotherapy. Moreover, trained immunity offers a novel perspective and strategic approach to optimizing treatment outcomes while concurrently minimizing treatment-related risks.

Sepsis is a systemic inflammatory disorder characterized by a dynamic inflammatory state that can manifest as both excessive inflammation and immunosuppression (197). Immune dysfunction serves as a pivotal mechanism underlying sepsis, encompassing abnormalities in both the innate and adaptive immune responses. In recent years, a variety of immune conditioning treatments have entered clinical trials, often aimed at modulating the body’s immune response to ameliorate the symptoms of sepsis. The concept of trained immunity has emerged, highlighting the immune system’s capacity for memory. This memory enables innate immune cells to elicit robust and non-specific responses upon exposure or re-exposure to antigens/infections or vaccines, thereby enhancing resistance against unrelated pathogens or diminishing the severity of infections (198). This attribute can be harnessed to prime the immune system in advance to mount a rapid and effective response upon encountering sepsis, through the development of specific immunomodulators or vaccines. Such an approach holds the potential to rectify the immune imbalance caused by sepsis, restore the normal function of the immune system, and improve patient survival rates. Expectantly, this may lead to a novel breakthrough in the clinical immunotherapy of sepsis.

## 7 Conclusion

Trained immunity, as a mechanism to augment myeloid cell function upon subsequent exposure to antigenic stimuli, may offer novel therapeutic approaches for diseases characterized by immunosuppression, such as sepsis or cancer, or contribute to the pathogenesis of autoinflammatory or autoimmune disorders. Consequently, the implications of trained immunity in the initiation or resolution of disease warrant comprehensive investigation. Given the heterogeneous nature of sepsis, individuals exhibit diverse responses to the condition, complicating treatment approaches and significantly impacting medical progress. Therefore, the diagnosis and management of sepsis face significant challenges, necessitating ongoing efforts to discover innovative treatment modalities. Trained immunity represents a non-specific intervention that can sustain immune system functionality amidst infection, bolster sustained immune responses, and support the body’s defense against pathogens.

In this review, we concentrate on the immunotherapeutic potential of trained immunity in sepsis, with the goal of elucidating novel therapeutic strategies for the management of this condition. Currently, the investigation into trained immunity in sepsis is nascent, with insufficient depth to fully comprehend its implications. However, studies using various mouse models of sepsis have unequivocally demonstrated that the activation of trained immunity modulates immune regulation, enhances the function of immune cells, fosters anti-inflammatory and anti-infective responses, and improves survival outcomes. Nonetheless, additional empirical evidence is required to substantiate the application of trained immunity in sepsis clinical trials. The regulatory impact of trained immunity on the immune system holds promise for the development of efficacious therapeutic interventions for sepsis. Broader, more profound, and systematic inquiries are necessary to fully appreciate the precise role and operational mechanism of trained immunity in sepsis. Such insights are particularly crucial for the personalized treatment of sepsis patients, ensuring optimal clinical efficacy.
